# Physicochemical and Biological Characterization of Fucoidan from *Fucus vesiculosus* Purified by Dye Affinity Chromatography

**DOI:** 10.3390/md14040079

**Published:** 2016-04-15

**Authors:** Ahmed Zayed, Kai Muffler, Thomas Hahn, Steffen Rupp, Doris Finkelmeier, Anke Burger-Kentischer, Roland Ulber

**Affiliations:** 1 Institute of Bioprocess Engineering, University of Kaiserslautern, Gottlieb-Daimler-Straße 49, 67663 Kaiserslautern, Germany; zayed@mv.uni-kl.de; 2 Department of Pharmacognosy, Tanta University, College of Pharmacy, El Guish Street, 8130 Tanta, Egypt; 3 Department of Life Sciences and Engineering, University of Applied Sciences Bingen, Berlinstraße 109, 55411 Bingen, Germany; K.Muffler@fh-bingen.de; 4 Department of Molecular Biotechnology, Fraunhofer Institute for Interfacial Engineering and Biotechnology, Nobelstraße 12, 70569 Stuttgart, Germany; thomas.hahn@igb.fraunhofer.de (T.H.); steffen.rupp@igb.fraunhofer.de (S.R.); doris.finkelmeier@igb.fraunhofer.de (D.F.); anke.burger-kentischer@igb.fraunhofer.de (A.B.-K.)

**Keywords:** fucoidan, toluidine blue O, anticoagulant, antiviral, dye-affinity chromatography

## Abstract

A comparative study concerning the physicochemical, monomeric composition and biological characters among different fucoidan fractions is presented. Common purification techniques for fucoidan usually involve many steps. During these steps, the important structural features might be affected and consequently alter its biological activities. Three purified fractions were derived from *Fucus vesiculosus* water extract which, afterwards, were purified by a recently-developed dye affinity chromatography protocol. This protocol is based on dye-sulfated polysaccharide interactions. The first two fractions were obtained from crude precipitated fucoidan at different pH values of the adsorption phase: pH 1 and 6. This procedure resulted in fucoidan_1 and 6 fractions. The other, third, fraction: fucoidan_M, however, was obtained from a buffered crude extract at pH 1, eliminating the ethanol precipitation step. All of the three fractions were then further evaluated. Results revealed that fucoidan_M showed the highest sulfur content (S%), 12.11%, with the lowest average molecular weight, 48 kDa. Fucose, galactose, and uronic acid/glucose dimers were detected in all fractions, although, xylose was only detected in fucoidan_1 and 6. In a concentration of 10 µg·mL^−1^, Fucoidan_6 showed the highest heparin-like anticoagulant activity and could prolong the APTT and TT significantly to 66.03 ± 2.93 and 75.36 ± 1.37 s, respectively. In addition, fucoidan_M demonstrated the highest potency against HSV-1 with an IC_50_ of 2.41 µg·mL^−1^. The technique proved to be a candidate for fucoidan purifaction from its crude extract removing the precipitation step from common purification protocols and produced different fucoidan qualities resulted from the different incubation conditions with the immobilized thiazine toluidine blue O dye.

## 1. Introduction

Fucoidan is a class of biopolymers that has a sulfated homo- or hetero-polysaccharide backbone. It is particularly found in the fibrilar part of the cell wall and intercellular spaces, especially of the brown seaweeds (Phaeophyta) [1]. The exact function of fucoidan is still not completely understood and needs more investigation, but it is assumed that it prevents the desiccation of the thallus tissues, especially when the algae is subjected to lower tide levels or in the higher temperature of summer [2]. It also acts as cross-linker between cellulose and hemicellulose and participates in the cellular structural integrity [3].

l-Fucose represents the major repeating monomer, that is linked by different glycosidic linkages (e.g., alternating α-1,3 and α-1,4 l-fucopyranosyls in fucoidan derived from *F. vesiculosus*) to form a straight or branched-chain polysaccharide skeletons. Other sugar monomers such as galactose, mannose, glucose, and xylose, as well as traces of uronic acids, could be present, in addition to sulfate and acetate ester groups, which bind in a species-dependent pattern [4,5].

The first extraction and isolation was carried out by Kylin approximately 100 years ago in 1913 [6]. Since then fucoidan research has been dramatically increased, especially during the last four decades, due to its promising and diverse pharmacological activities (e.g., antiviral, antithrombotic and anticoagulant, anti-inflammatory, cytotoxic, and their effects against various renal and hepatic diseases) [7].

Fucoidan is freely soluble in solvents of higher dielectric constants like water, due to isolated shielded opposite groups [8], and that is why hot water extraction, either in acidic or alkaline conditions, is usually the method of choice for its extraction. However, solvents of lower dielectric constants, like ethanol, are usually used for precipitation and isolation from other co-extracted natural compounds.

Different methods (e.g., hot acidic or alkaline, enzyme-, microwave- or ultrasound-assisted aqueous extraction) were developed to optimize the fucoidan extraction process. Nevertheless, time-consuming and expensive purification techniques are still applied in order to obtain a purified grade from its crude extracts. Among applied purification techniques are anion exchange and gel-permeation chromatography, depending on its higher anionic charge density and molecular weights.

Anion exchange chromatography has several disadvantages such as adsorption of other acidic compounds in alkaline pH such as alginate or polyphenols or even to free hydrolyzed sulfate ester groups of fucoidan, which elute afterwards in desorption step with intact fucoidan and distort the accurate determination of sulfur content (S%). In addition, positively-charged quaternary ammonium groups of resin beads could be leaked to give another possibility for contamination and higher nitrogen content (N%). The resin usually needs a tedious loading regeneration step after each cycle to regain its functional groups and capacity. Major structural features of fucoidan, such as molecular weight, monomeric composition, sulfate ester content, and position, are affected during extraction and purification processes and resulted in inaccurate characters [9]. Additionally, seasonal and geographical factors are also involved in such structural conflicts [10].

Recently, a purification protocol was developed based on dye affinity chromatography and proved its ability to capture intact fucoidan selectively from synthetic and crude extracts by immobilized thiazine dyes e.g., toluidine blue and thionine acetate. This protocol was performed through several steps of washing of the derivatized beads with different solvents to remove non-specific binding compounds before the elution step to obtain a high purified grade [11].

The present research aims to apply this new protocol using different conditions e.g., pH and simultaneous extraction and purification to obtain several fucoidan fractions from the brown macroalgae *Fucus vesiculosus*, in addition to their evaluation using physicochemical characters and monomeric composition, as well as biological activities, such as anticoagulant and antiviral activities.

## 2. Results

### 2.1. Extraction and Preparation of Crude Extract

Fucoidan represents a variable constituent in brown algae and usually affects by seasonal and geographical factors [12]. In *F. vesiculosus*, only 12% *w*/*w* of the dry biomass represents fucoidan content [13]. Therefore, an intensive pretreatment procedure should be performed to remove the other natural compounds (e.g., mannitol, lipids, polyphenols, *etc*.), which could be co-extracted or interfered afterwards with purification processes [14,15]: acetone removed chlorophyll, pigments, and fatty acids, whereas a hexane:isopropanol mixture removed lipids and more polar fatty acids. An 80% (*v*/*v*) ethanol was used for removing of the major reserve food material mannitol for the algae. An ethanol/water/formaldehyde mixture at pH 2 was used to polymerize polyphenols, which are usually tightly bound to fucoidan and responsible for the brown color of contaminated fucoidan [16], and finally 80% (*v*/*v*) ethanol was used again for washing and cleaning up the biomass from residual formaldehyde and polyphenol complex.

### 2.2. Purification of Fucoidan from Crude Extract

Depending on results described by Hahn *et al.* [17], the interaction between toluidine blue and fucoidan is strong enough and not only driven by ionic interaction, but also by disperse interactions between the stacked dye molecules. These results were downstream processed and applied to purify fucoidan at different pH values: 1 and 6 from its crude extract, as well as in a single step by simultaneous extraction and purification in maleic acid buffer (MAB) at pH 1 without a precipitation step. This highly-acidic buffered pH was preferred to help acidic compounds carrying groups of lower p*K_a_* especially sulfate ester groups of fucoidan to be in ionized form and captured. Interestingly, this technique overcame also other critical economic and environmental issues of purification such as removal of precipitation step with ethanol or other cationic surfactants e.g., cetyl trimethyl ammonium bromide (CTAB). Like other common techniques, different fractions could be also obtained by applying different NaCl molarities in the elution step.

### 2.3. Evaluation of the Beads Leakage and Dialysis Process

Dextran from *Leuconostoc* sp. (Mr ~500 kDa) was chosen due to its negligible affinity or interaction with the immobilized dye and also for the absence of S and N, which are present in the MES buffer used in the elution and dye structures. Incubation of the dextran solution with the beads at the same conditions of the fucoidan elution step confirmed the absence of any kind of contamination either from the immobilized dye or residuals of eluent by results of elemental analysis of dextran before and after incubation. Results after incubation showed similar C% and H% content with the absence of N and S atoms. Table 1 clarifies elemental analysis (CHNS) of dextran from *Leuconostoc* sp. (Mr ~500 kDa) before and after incubation with the derivatized beads.

### 2.4. Physicochemical Investigations

#### 2.4.1. Appearance and Solubility

White, fluffy, and hygroscopic powders were obtained as fucoidan_1 and fucoidan_6, while fucoidan_M was buff-colored. All of them were soluble in water, sparingly soluble in dimethyl sulfoxide (DMSO), and insoluble in ethanol.

#### 2.4.2. Elemental Analysis (CHNS Analysis)

As shown in Table 2, the presence of traces of nitrogen content (N%) in all fucoidan types, in contrast with reference fucoidan, suggested the presence of some amino-containing compounds (e.g., protein or amino sugars) [9]. Fucoidan_M demonstrated the lowest N%: 0.24% (*m*/*m*) compared with the other fractions: fucoidan_1 and fucoidan_6. Nevertheless, it showed the highest S%: 12.11% (*m*/*m*), which is critical for some biological activities (e.g., antiviral activity). As a result, sulfate ester content could be calculated using the ratio between sulfur atom and sulfate ester masses: 2.99582. Therefore, the sulfate ester content in fucoidan_M was 36.28% (*w*/*w*), while in commercial fucoidan 26.6%.

In terms of sulfate groups per each monosaccharide unit, each fucose unit binds to, approximately, one sulfate ester group in fucoidan_M molecule. In fact, this opposes fucoidan model hypothesized by Cumashi *et al.* [18] who concluded that there are two sulfate ester groups per three fucose units. So, this procedure was able to protect the sulfation ester pattern from hydrolysis to a great extent. Hence, it can be used to determine fucoidan native sulfate content.

#### 2.4.3. Melting Point

As the melting point is affected by impurities and molecular weight, melting points are used for more characterization and proof relations with molecular weight measurement [19]. All organic polymers—such as fucoidan—melt and decompose by heat exposure. All fucoidan fractions behave by the same sequence with increasing the temperature; namely, changing color: yellow, brown, and then to black, and is reflected by gas development. The temperature ranges between the start of melting and decomposition points were 5–6 °C for fucoidan_1 and fucoidan_M, while it was wide (20 °C) for fucoidan_6. Fucoidan_6 proved its higher molecular weight measurement by showing the highest melting point. Table 3 summarizes a comparison among the phases at which the different fractions of fucoidan changed their color, melted, and then decomposed with charring at higher temperatures.

#### 2.4.4. Specific Optical Rotation [α]^22^_589_

Specific optical rotation results, as shown also in Table 3, proved the levorotatory (*l*) and asymmetric nature of all fucoidan fractions. This also confirmed the presence of l-fucose as a major stereoisomer of fucose monomer. [α]^22^_589_ for all fucoidan fractions, including the reference analogue were similar and the values were in the range from −117° for fucoidan_6 to −130° for fucoidan_M. These values could be related also to fucoidan molecular weight and fucose content. The values are also consistent with values reported previously: −123° [7].

#### 2.4.5. Molecular Weight Averages (Mw, Mp, Mn)

Weight-average molecular weight (Mw), peak molecular weight (Mp), and number-average molecular weight (Mn) were measured to assess the polymer chain length characteristics and evaluate the effect of the applied different extraction and purification conditions. Fucodian_M had the lowest values of Mw, Mp, and Mn with 48, 18, and 26 kDa, respectively, in comparison with other fucoidan fractions [11], proposing a partial acid hydrolysis of the polysaccharides during extraction and purification processes [11]. Lower molecular weight fucoidan (LMWF) could be advantageous to improve the diffusion rates of fucoidan and hence decreasing the time required for adsorption phase, in addition to its biological importance e.g., proangiogenetic activity [20]. Effect of NaCl molarity was also studied on fucoidan elution % and relations with molecular weight averages. As shown in Table 4, increasing of NaCl molarity to 3 M resulted in increasing the % elution and higher molecular weight of fucoidan fractions obtained. Table 4 summarizes molecular weight averages of fucoidan fractions after incubation of crude extract at pH 1 and eluted in relation with different NaCl molarities: 1, 2, and 3.

### 2.5. Monomeric Composition

As monomeric composition is one of the critical factors affecting biological activities, it was determined for all fractions. As Table 5 shows, fucose is the major monomer in all fucoidan fractions and represents more than 80%. Other monomers, such as galactose and uronic acid/glucose dimers, were also detected; however xylose was detected only in fucoidan_1 and fucoidan_6. Uronic acids/glucose dimers were only detected by MS; nevertheless, their quantity could not be accurately determined since there were no standards available in addition to their poor retention behavior.

### 2.6. Spectroscopical Investigation

**Figure 1:**
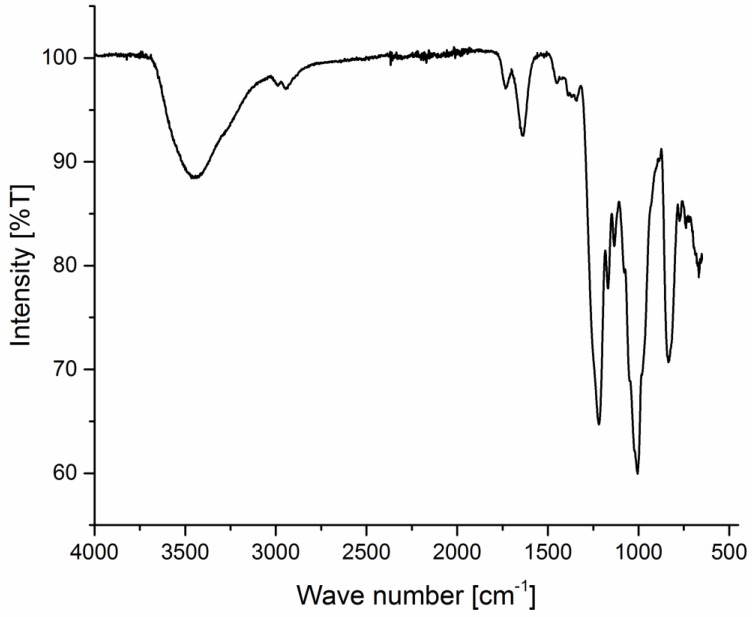
FTIR spectrum of fucoidan_M.

FT-IR spectroscopy was performed to identify fucoidan major characteristic peaks, compare the similarity with its reference analogue, and assess the effect of extraction and purification conditions on the chemical structure, especially in the case of fucoidan_M. All spectra showed comparable homology to the reference analogue. With fucoidan_M, as an example, FTIR spectrum and its characteristic peaks were identified and interpreted thoroughly as shown in Figure 1: a broad peak at 3465 cm^−1^ for OH group of monosaccharide monomer, 2984 and 2940 cm^−1^ for the aliphatic C–H, a peak at 1730 cm^−1^ for C=O stretching vibration for acetate groups, the peak at 1635 cm^−1^ was represented for O–C–O stretching vibration, 1218 cm^−1^ for asymmetric stretching vibration of S=O of the sulfate group, 1005 cm^−1^ for the ether bond C–O, 835 cm^−1^ for C–S–O, and 667 cm^−1^, which is a characteristic band for dexoysugars as fucose. FTIR revealed also a complex pattern of substitution at C-4 position by demonstrating a sharp band at 835 cm^−1^ (S=O) and a shoulder at 815 cm^−1^ (C–S–O).

All of these data were consistent with fucoidan’s chemical structure, proposed by Cumashi *et al.* [18] and confirmed the negligible effect of the extraction and purification technique on the fucoidan chemical structure.

### 2.7. Biological Investigations

#### 2.7.1. Anticoagulant Activity

##### Activated Partial Thromboplastin Time (APTT)

**Figure 2:**
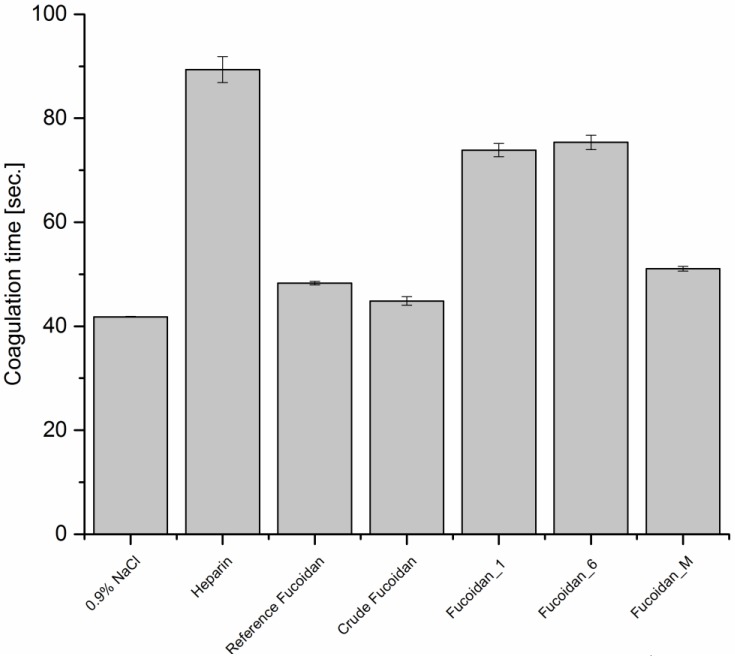
APPT of different types of fucoidan at a concentration of 10 µg·mL^−1^ using 0.9% NaCl and 5 µg·mL^−1^ as negative and positive control, respectively (*n* = 3).

Blood coagulation is initiated through either intrinsic or extrinsic pathway. The two pathways merge at the common point of prothrombin conversion to thrombin and proceed afterwards to form fibrin threads. APTT evaluates mainly the effect of anticoagulants on the intrinsic pathway of blood coagulation system. Figure 2 shows that the purification process improved the anticoagulant activity of crude fucoidan through increasing APTT, where fucoidan_1 and 6 increased coagulation times to 73 and 75 s, respectively, in comparison with only 44.8 s for non-purified crude fucoidan. Fucoidan_M increased coagulation time, as well, but to a lesser extent: 51 s. This was nearly similar to the reference analogue: 48.3 s. These prolongations were significant in comparison with negative control (0.9% NaCl) which recorded 41.8 s.

##### Prothrombin Time (PT)

**Figure 3:**
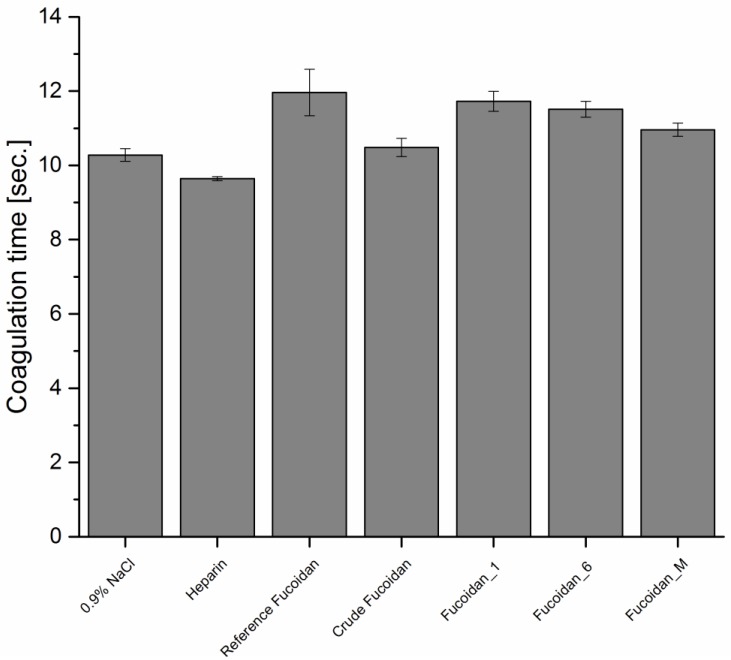
PT of different types of fucoidan at a concentration of 10 µg·mL^−1^ using 0.9% NaCl and 5 µg·mL^−1^ as negative and positive control, respectively (*n* = 3).

PT is usually used to evaluate the effect of anticoagulants on the blood extrinsic coagulation pathway. It is obvious in Figure 3 that, all types of fucoidan were not able to prolong PT significantly in comparison with the negative control. These were consistent with the mechanism of fucoidan anticoagulant activity which is similar to heparin and mediated principally by inhibition of the serine protease thrombin enzyme and/or heparin cofactor II [21].

##### Thrombin Time (TT)

**Figure 4:**
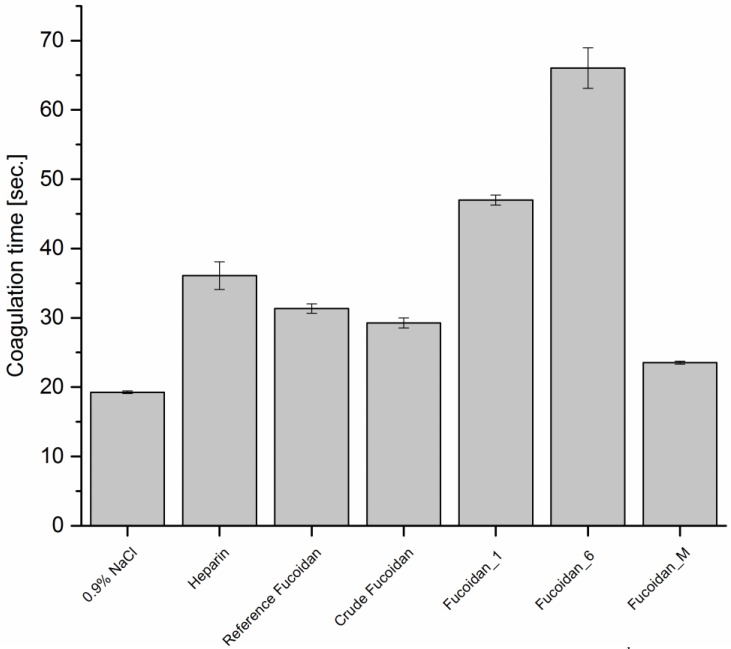
TT of different types of fucoidan at a concentration of 10 µg·mL^−1^ using 0.9% NaCl and 5 µg·mL^−1^ as negative and positive control, respectively (*n* = 3).

TT measures specifically the effect of anticoagulants against thrombin, which catalyzes the step of fibrinogen conversion to fibrin threads in the last steps of clot formation. Results, as shown in Figure 4, revealed a similar pattern as in Figure 2, where fucoidan_6 also showed the highest activity. These similarities suggested a correlation between APTT and TT and increasing in APTT might result from, and a consequence of, inhibition of enzymes and cofactors of common pathway more than the intrinsic pathway. Fucoidan_1 and 6 prolonged coagulation time significantly to 47 and 66 s, respectively, compared with 19.27 s for the negative control, while fucoidan_M showed the weakest effect on coagulation time. It increased the coagulation time only to 23.5 s compared with 31.3 and 29.2 s for reference and crude fucoidan, respectively. Even with higher concentrations (e.g., 50 µg·mL^−1^), a non-linear and small effect on the coagulation time were observed and increasing it to 31.1 s. These differences between fucoidan fractions relate to the polysaccharide chain, molecular weight, and structure comfortability, where an enough long sugar chain and comfortable structure are required even more than sulfate content for thrombin deactivation [7]. The lower effect of fucoidan_M on TT may be also due to its higher sulfate content which may be increased over the threshold value and led to a decrease in the anticoagulant activity [22,23]. Nevertheless, not all fucoidan extracted from different brown algae has the same antithrombin activity and effect on TT due to fucoidan endogenesis restraints [24,25].

#### 2.7.2. Antiviral Activity

**Figure 5:**
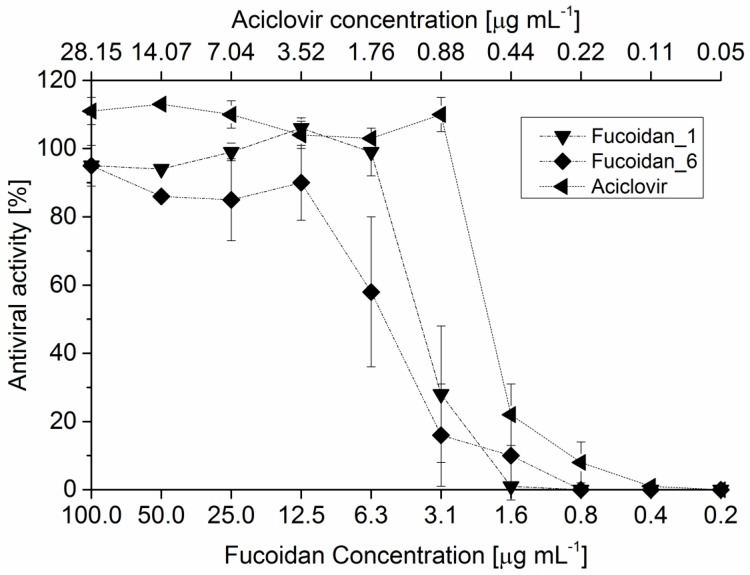
Comparison between antiviral activities against HSV-1 of different types of fucoidan at a different serial dilution using aciclovir as a positive control. Reference, crude, and fucoidan_M are not shown to give a concise and simple overview.

Fucoidan exhibits its antiviral activity against HSV-1 mainly through inhibition of viral sorption and penetration through cellular surface modification and, subsequently, prevent the viral syncytium formation [26]. For this action, sulfate ester group position is very important and critical in comparison with the anticoagulant activity. The fraction with the highest sulfate content, fucoidan_M, exerted a highly potent antiviral effect in comparison with other types. Figure 5 demonstrated the antiviral activity (%) with different serial dilution for different fucoidan fractions against HSV-1 and IC_50_ values were also calculated, as shown in Table 6. IC_50_ varied among the different fractions from 2.41 µg·mL^−1^ for fucoidan_M to 5.69 µg·mL^−1^ for fucoidan_6. Interestingly, fucoidan is has the advantage of its lower cytotoxicity than clinically-used antiviral drugs, such as acyclovir.

## 3. Experimental Sections

### 3.1. Chemicals

Sepabeads^®^ EC-EA (S-grade) was a gift from Resindion s.r.l. (Binasco MI, Italy). Particle size range of the carrier was 100–300 µm with a median pore diameter of 10–20 nm. Reagents used to measure the anticoagulant activity were purchased from Siemens Healthcare Diagnostics Products GmbH (Marburg, Germany). All other chemicals including reference fucoidan isolated from *F. vesiculosus* (≥95% pure) were purchased from Sigma Aldrich^®^ (St. Louis, MO, USA).

### 3.2. Instrumentations

HPLC pump L 7100 with an auto sampler AS-2000 A (both Merck-Hitachi, Darmstadt, Germany), a GPC_MCX column 8 × 30 mm (PSS, Mainz, Germany) and Shodex^®^ RI-101 detector (Shimadzu Corporation, Kyoto, Japan) were used for molecular weight averages measurement. Spectrum 100 FT-IR, (Perkin Elmer, Waltham, MA, USA) was used to identify and characterize fucoidan fractions in comparison with reference fucoidan. An overhead shaker Multi Bio RS-24 and a thermoshaker TS-100 (Biosan, Riga, Latvia) were used to stir suspensions during adsorption and elution incubation periods of purification process, respectively. Elemental analysis was performed using Vario Micro cube WLD Board V 2.0.11 (Elementar Analysensysteme GmbH, Hanau, Germany). P-2000 digital polarimeter (Jasco, Gross-Ulmstadt, Germany) supplied with a sodium spectral lamp and adjusted at λ 589 nm for specific optical rotation measurement. DigiMelt-MPA 160, SRS (Scientific Instruments GmbH, Gilching bei München, Germany) for melting point determination. A conductivity meter set (Qcond 2200, VWR International GmbH, Darmstadt, Germany) was used for conductivity measurement. A BCS^®^ System (Siemens Healthcare Diagnostics Products GmbH, Marburg, Germany) was programmed to perform the different coagulation protocol and measure the coagulation times. Fluorescence measurements were performed using a Spectra-Photometer V630 (Jasco, Gross-Ulmstadt, Germany) to assess the antiviral activity.

### 3.3. Extraction and Preparation of the Crude Extract

#### 3.3.1. Algal Preparation and Pretreatment

Fresh algal biomass of *F.* *vesiculosus* was harvested from the North Sea at the region of south beaches of Wilhelmshaven (Germany, 53°31.236N, 8°13.849E) in July 2007. The algal biomass was washed with tap, and then deionized, water, air-dried for two days, then in the oven at 50 °C until constant weight, and milled afterwards. 100 g dried algal powder, before the extraction step, were handled by several pretreatment steps successively under continuous stirring overnight at room temperature with 1 L of acetone, hexane:isopropanol mixture (3:2), 80% (*v*/*v*) ethanol, ethanol:water:formaldehyde (80:15:5) at pH 2, and finally washed again with 1 L 80% (*v*/*v*) ethanol. After each step, the suspension was centrifuged (4000 rpm, 10 min), and the supernatant is decanted. The pretreated algal powder was then dried again at 50 °C and stored at room temperature in a well-closed plastic container.

#### 3.3.2. Extraction

Two methods for fucoidan extraction were applied:

**Procedure A:** The first method was performed by exhaustion of 10 g of pretreated powder with 100 mL 1% (*w*/*v*) aqueous CaCl_2_ for 6 h at 70 °C under reflux with continuous stirring at pH 2, as described previously by Hahn T. *et al.* [11]. After centrifugation (4500 rpm, 15 min), the algal biomass was removed and the supernatant was neutralized by 0.2 M ammonium carbonate. Crude fucoidan was then isolated via precipitation by ethanol with a final concentration of 70% (*v*/*v*), cooling overnight at 4 °C, centrifugation, and then drying of the precipitate at 50 °C.

**Procedure B:** The other method administered 20 mM maleic acid buffer (MAB) at pH 1 as a solvent for the 1% (*w*/*v*) CaCl_2_ solution. The pretreated algal powder was afterwards extracted using the same conditions previously mentioned in Procedure A. The pH was adjusted regularly every 2 h at 1 by 1 M HCl, as necessary. Centrifugation (4500 rpm, 15 min) was also used to separate the supernatant from the algal biomass. The supernatant was stored afterwards in the refrigerator untl the next step of purification.

#### 3.3.3. Immobilization of Toluidine Blue, Purification, and Recovery of Fucoidan Fractions

According to immobilization protocol described by HahnT, *et al.* [11], an aqueous 2 mM toluidine blue O (TB) solution was prepared and immobilized on the Sepabeads^®^ EC-EA to produce the derivatized beads.

The first two fucoidan fractions were obtained by reconstitution of crude fucoidan powder obtained by procedure A in a concentration of 2.5 mg·mL^−1^ in two different buffer solutions: 20 mM MAB pH 1 and 20 mM MES pH 6 and purified to obtain fucoidan_1 and fucoidan_6, respectively. Procedures for purification and recovery were performed by incubation of 50 mg of the derivatized beads with 1.5 mL of the stock solutions in a 2 mL reaction vessel at room temperature for 40 h and placed in an overhead shaker adjusted at 60 rpm. The beads were then washed with water and 0.1 M NaCl in 20 mM MAB at pH 2, successively. 1.5 mL of 3 M NaCl dissolved in 30% (*v*/*v*) ethanol in a 20 mM MES buffer pH 6 was used as an eluent with shaking (800 rpm) at 50 °C. The eluates were afterwards dialyzed through a MWCO 3.5 kD membrane tube for salt removal and lyophilized at the end.

The third fraction, fucoidan_M, was isolated by incubation of 75 mg of derivatized beads with 1.5 mL of crude extract obtained by procedure B for 48 h. The other steps for washing and recovery were performed as described previously with the other two fractions.

#### 3.3.4 Evaluation of Beads Leakage

Dextran solution of a concentration 2.5 mg·mL^−1^ from *Leuconostoc* sp. (Mr ~500 kDa) was prepared in the same eluent of fucoidan (3 M NaCl in 30% ethanol in 20 mM MES pH 6) and incubated with the derivatized beads at conditions mimic to that applied in fucoidan elution phase for 12 h. The solution obtained after incubation was afterwards dialyzed, lyophilized, and elementally analyzed in comparison with non-treated dextran powder.

### 3.4 Physicochemical Investigations

#### 3.4.1 Molecular Weight Averages

Molecular weight measurements were performed using an isocratic HPLC pump L 7100 with an auto sampler AS-2000 A. Samples were dissolved in eluent in a concentration of 4 mg·mL^−1^ and mixed with an equal volume of 1 mg·mL^−1^ ethylene glycol solution as a flow marker. Separation was performed at 25 °C using a GPC_MCX column 8 × 30 mm, which was previously calibrated with dextran sulfate (analytical standard grade for GPC) of different molecular sizes (25–670 kDa). The injection volume was 10 µL and eluent was 0.05 M phosphate buffer at pH 9.1 with a volumetric flow rate of 1 mL·min^−1^. The sample detections were performed using Shodex^®^ RI-101 detector.

#### 3.4.2. Melting Point

Two mg of each fucoidan fraction was placed in a capillary tube and placed in the melting point apparatus. Temperature increment was 2 °C·min^−1^ and three temperature points, at which powders started to melt, changed their color, and finally decomposed with charring, were observed and recorded.

#### 3.4.3. Specific Optical Rotation

An aqueous 0.4% *w*/*v* solution of each fucoidan was prepared and the specific optical rotation was measured at 22 °C.

### 3.5. Monomeric Composition

Monomeric composition of the different fractions of fucoidan was determined according to protocols developed by Rühmann, *et al.* [27]. Briefly, 1 g·L^−1^ aqueous solution of the three fucoidan fractions were prepared and incubated with 2 M trifluroacetic acid for 90 min at 121 °C. The monomers fucose, xylose, and galactose of hydrolyzed polysaccharides were detected by UV–IS, while glucose and uronic acid dimers were detected by MS.

### 3.6. Biological Investigations

#### 3.6.1. Anticoagulant Activity

##### Activated Partial Thromboplastin Time (APTT)

APTT was determined for all fucoidan fractions, according to the protocol described by Anderson *et al.* [28] with few modifications. All the samples were prepared in an isotonic solution of 0.9% *w*/*v* NaCl. The device was programmed to mix 0.6 mL of platelet-poor plasma with 0.3 mL of 10 µg·mL^−1^ fucoidan solution. Each mixture was then incubated for 60 s at 37 °C before the addition of 0.5 mL pre-warmed APPT reagent and then incubated again for 5 min at 37 °C. Afterwards, 0.6 mL of a pre-warmed 0.25 M CaCl_2_ solution was added and the time for clot formation was recorded. These steps were repeated three times. Heparin in a concentration of 5 µg·mL^−1^ and 0.9% *w*/*v* NaCl were used as positive and negative control, respectively.

##### Prothrombin Time (PT)

PT was also determined using the protocol of Quick [29] with some modifications. The device was also programmed to perform the same procedure mentioned previously in APTT determination, but each poor-platelets plasma and fucoidan solution mixture was incubated for 3 min at 37 °C. Afterwards, 0.6 mL pre-warmed PT reagent was added and the time for clot formation was observed and repeated three times. Heparin and NaCl were also used as positive and negative control, respectively.

##### Thrombin Time (TT)

TT was determined applying protocol described by Denson and Bonnnar [30]. In a ratio of 3:1, 0.6 mL of platelet-poor plasma was mixed with 0.2 mL of fucoidan solutions at a concentration of 10 µg·mL^−1^. Each mixture was then incubated for 3 min at 37 °C before the addition of 0.2 mL pre-warmed TT reagent and the time for clot formation was recorded with three repetitions. Heparin (5 µg·mL^−1^) and NaCl (0.9% *w*/*v*) were used as positive and negative control, respectively.

#### 3.7. Antiviral Assay

The antiviral activity was carried out against a representative of the double strand DNA (*ds*DNA) viruses: Herpes Simplex virus-type 1 (HSV-1). The screening assay was based on Tissue Culture Infection Dose 50 (TCID50) for antivirals and modified by Kleymann and Werling [31]. A stock solution of 5 mg·mL^−1^ of the three different fucoidan fractions were dissolved in phosphate saline buffer (PBS) at pH 7.4, while acyclovir as 10 mM in DMSO. The 50% inhibitory concentration (IC_50_) was determined using a two-fold serial dilution (10 different concentrations) in the range of 0.2–100 and 0.054–28 µg·mL^−1^ for fucoidan solutions and acyclovir, respectively. Each well contained 10,000 cells of Vero B4 cells (50 µL of a solution with 200,000 cells/mL provided in Roswell Park Memorial Institute medium (RPMI) containing 10% fetal calf serum (FCS) and penicillin/streptomycin (PS)), 50 µL pathogen with 5 to 500 CFU of HSV1 (strain HF ATCC-VR-260), the final volume of each well was then completed to 200 µL by the culture medium. Negative controls are performed by mixing 50 µL of pathogen, 50 µL cell suspensions, and 100 µL medium. After the respective incubation period, the wells of the microplate were washed with phosphate-buffered saline (PBS, 200 μL) and then filled with 200 μL PBS containing 10 μg·mL^−1^ fluorescein diacetate (FDA), which is widely used to count viable cells and analyzes their vitality. Viable cells are able to convert the dye enzymatically by a cellular esterase activity releasing the fluorophore from the quenched dye of the non-fluorescent FDA. After 45 min of incubation at room temperature, fluorescence (RFU, relative fluorescence units) was measured using 485 nm for excitation and 538 nm for emission.

## 4. Conclusions

Physicochemical and biological evaluation of different fucoidan fractions purified by dye affinity chromatography is presented for the first time. These fractions were achieved by a recently-developed dye affinity chromatography technique, which is based on a specific formation of a charge transfer complex between thiazine dyes and sulfated polysaccharides. In addition, development of this technique helped downstream processing of extraction and purification simultaneously through selective capture of fucoidan from its crude extract, obtaining a new fraction of fucoidan called fucoidan_M. Pharmacological comparison between the different fucoidan fractions showed the importance of chain length, molecular weight, and structure comfortability for the anticoagulant activity, while sulfate ester groups position and content for the anti-herpetic activity.

Production of different fucoidan fractions with these qualities is a promising step on the way to standardize a method for fucoidan extraction, reveal new secrets of fucoidan structure-activity relationship (SAR) and elucidate its native and diverse chemical structures. It also helps to provide the market with a protocol for a reproducible commercial production of fucoidan.

## Figures and Tables

**Table 1:** Elemental analysis (CHNS) results of dextran from *Leuconostoc* sp. (Mr ~500 kDa) before and after incubation with derivatized beads under the same conditions of the fucoidan elution step. The table also shows ±SD (standard deviation) of the used reference standard: acetanilide.

Sample	N (%) ± 0.02 *	C (%) ± 0.06	H (%) ± 0.1	S (%) ± 0.00
Dextran	n.d. **	40.00	6.53	n.d.
Dextran after incubation	n.d.	41.23	6.45	n.d.

* ±SD of used reference standard acetanilide; ** n.d.: not detected.

**Table 2:** Elemental analysis (CHNS) results of fucoidan_M compared with commercially available pure analogue. The table also shows SD of the reference standard used: sulfanilic acid.

Sample	N (%) ± 0.03	C (%) ± 0.04	H (%) ± 0.01	S (%) ± 0.06
Reference Fucoidan ^1^	n.d. *	24.78	4.42	8.89
Fucoidan ^2^	0.3	24.14	4.43	11.18
Fucoidan ^3^	0.34	26.12	4.63	9.83
Fucoidan_M ^4^	0.26	23.31	4.28	12.11

^1^ Fucoidan (≥95% pure isolated from *F. vesiculosus*) purchased from Sigma-Aldrich^®^; ^2^ purified fraction from crude fucoidan at pH 1; ^3^ purified fraction from crude fucoidan at pH 6; ^4^ purified fraction extracted and captured from MAB crude extract at pH 1; * not detected.

**Table 3:** Comparison between the different fractions, regarding start, color change, and decomposition temperature points, as well as specific optical rotation [α]^22^_589_.

Sample	Start (°C)	Color Change (°C)	Decomposition (°C)	Specific Optical Rotation [α]^22^_589_
Reference Fucoidan ^1^	- *	-	-	−121
Fucoidan_1 ^2^	130	132–133	135	−128
Fucoidan_6 ^3^	140	153–156	161	−117
Fucoidan_M ^4^	130	133–134	136	−130

^1^ Fucoidan (≥95% pure isolated from *F. vesiculosus*) purchased from Sigma-Aldrich^®^; ^2^ purified fraction from crude fucoidan at pH 1; ^3^ purified fraction from crude fucoidan at pH 6; ^4^ purified fraction extracted and captured from MAB crude extract at pH 1; * not determined.

**Table 4:** Effect of eluent NaCl molarity on % fucoidan elution and molecular weight averages (Mw, Mn, and Mp) after an adsorption step at pH 1 for 40 h (*n* = 2).

Molarity of NaCl (M)	Adsorbed Fucoidan (%) ± SD	Eluted Fucoidan (%) ± SD	Molecular Weight Averages (×10^4^) Da		
			Mw	Mn	Mp
1	87.13 ± 0.008	42.4 ± 0.28	5.7	4.1	3.7
2		57.75 ± 0.49	6.6	4.8	4.9
3		70.3 ± 0.98	9.2	4.9	4.4

**Table 5:** % Monomeric composition of different fucoidan fractions. Uronic acids/glucose dimers are also detected in all fractions.

Composition	% Galactose	% Xylose	% Fucose
Fucoidan_1 ^1^	7.4 ± 0.06	4.0 ± 0.06	88.59 ± 0.03
Fucoidan_6 ^2^	8.99 ± 0.25	4.2 ± 0.45	86.8 ± 0.45
Fucoidan_M ^3^	7.56 ± 0.2	- *	92.43 ± 0.2

^1^ Purified fraction from crude fucoidan at pH 1; ^2^ purified fraction from crude fucoidan at pH 6; ^3^ purified fucoidan extracted and captured from MAB crude extract at pH 1; * not detected.

**Table 6:** Summary of IC_50_ (μg·mL^−1^) of different types of fucoidan isolated and purified from *F. vesiculosus* against HSV-1 in comparison with aciclovir.

	IC_50_ (μg·mL^−1^)
Reference Fucoidan ^1^	3.3
Fucoidan_1 ^2^	4.09
Fucoidan_6 ^3^	5.69
Fucoidan_M ^4^	2.41
Crude fucoidan	3.99
Acicolvir	0.52

^1^ Fucoidan (≥95% pure) isolated from *F. vesiculosus* purchased from Sigma-Aldrich^®^; ^2^ purified fraction from crude fucoidan at pH 1; ^3^ purified fraction from crude fucoidan at pH 6; ^4^ purified fucoidan extracted and captured from MAB crude extract at pH 1.
